# Supplementary material for: Machine Learning Accelerates Crystallization for Structure Determination
Source: Angew Chem Int Ed Engl. 2026 May 4;65(25):e1218503. doi: 10.1002/anie.1218503 (PMC13266958; doi:10.1002/anie.1218503)

checkCIF/PLATON report

Structure factors have been supplied for datablock(s) lcz065

THIS REPORT IS FOR GUIDANCE ONLY. IF USED AS PART OF A REVIEW PROCEDURE FOR PUBLICATION, IT SHOULD NOT REPLACE THE EXPERTISE OF AN EXPERIENCED CRYSTALLOGRAPHIC REFEREE.

No syntax errors found. CIF dictionary Interpreting this report

Datablock: lcz065

Bond precision: C-C = 0.0110 A Wavelength=1.54184

Cell:	a=9.3849(1)	b=12.7862(1)	c=32.9001(3)
	alpha=90	beta=90	gamma=90
Temperature:	100 K		

	Calculated	Reported
Volume	3947.92(6)	3947.92(6)
Space group	P 21 21 21	P 21 21 21
Hall group	P 2ac 2ab	P 2ac 2ab
Moiety formula	C34 H23 Ag3 F18 N6 O3	C15 H3 Ag3 F18 N6, C19 H20 O3
Sum formula	C34 H23 Ag3 F18 N6 O3	C34 H23 Ag3 F18 N6 O3
Mr	1229.20	1229.19
Dx,g cm-3	2.068	2.068
Z	4	4
Mu (mm-1)	13.028	13.028
F000	2384.0	2384.0
F000’	2394.97	
h,k,lmax	11,16,41	11,15,41
Nref	8415[4724]	7817
Tmin,Tmax	0.144,0.161	0.691,1.000
Tmin’	0.064	

Correction method= # Reported T Limits: Tmin=0.691 Tmax=1.000
AbsCorr = MULTI-SCAN

Data completeness= 1.65/0.93   Theta(max)= 77.805

R(reflections)= 0.0324(7399)   wR2(reflections)= 0.0694(7817)

S = 1.048   Npar= 589

The following ALERTS were generated. Each ALERT has the format
test-name_ALERT_alert-type_alert-level.
Click on the hyperlinks for more details of the test.

Alert level C

```
PLAT042_ALERT_1_C Calc. and Reported MoietyFormula Strings  Differ     Please Check
              Calc: C34 H23 Ag3 F18 N6 O3
              Rep.: C15 H3 Ag3 F18 N6, C19 H20 O3
PLAT090_ALERT_3_C Poor Data / Parameter Ratio (Zmax > 18) ........       7.84 Note
PLAT213_ALERT_2_C Atom F00W            has ADP max/min Ratio .....        3.9 prolat
PLAT213_ALERT_2_C Atom F014            has ADP max/min Ratio .....        3.9 prolat
PLAT220_ALERT_2_C NonSolvent   Resd 1  C   Ueq(max)/Ueq(min) Range        3.5 Ratio
PLAT220_ALERT_2_C NonSolvent   Resd 1  F   Ueq(max)/Ueq(min) Range        3.5 Ratio
PLAT234_ALERT_4_C Large Hirshfeld Difference C3       --C01H     .       0.16 Ang.
PLAT241_ALERT_2_C High   'MainMol' Ueq as Compared to Neighbors of       C01H Check
PLAT241_ALERT_2_C High   'MainMol' Ueq as Compared to Neighbors of       C01P Check
PLAT342_ALERT_3_C Low Bond Precision on  C-C Bonds ..............       0.011 Ang.
PLAT369_ALERT_2_C Long   C(sp2)-C(sp2) Bond  C00R     - C00X     .       1.55 Ang.
```

Alert level G

```
PLAT002_ALERT_2_G Number of Distance or Angle Restraints on AtSite          2 Note
PLAT003_ALERT_2_G Number of Uiso or U(i,j)  Restrained non-H-Atoms          1 Report
PLAT083_ALERT_2_G SHELXL Second Parameter in WGHT  Unusually Large      13.23 Why ?
PLAT142_ALERT_4_G s.u. on b - Axis Small or Missing .............    0.00010 Ang.
PLAT143_ALERT_4_G s.u. on c - Axis Small or Missing .............    0.00030 Ang.
PLAT172_ALERT_4_G The CIF-Embedded .res File Contains DFIX Records          1 Report
PLAT186_ALERT_4_G The CIF-Embedded .res File Contains ISOR Records          1 Report
PLAT242_ALERT_2_G Low    'MainMol' Ueq as Compared to Neighbors of       C016 Check
PLAT242_ALERT_2_G Low    'MainMol' Ueq as Compared to Neighbors of       C01C Check
PLAT242_ALERT_2_G Low    'MainMol' Ueq as Compared to Neighbors of       C01I Check
PLAT242_ALERT_2_G Low    'MainMol' Ueq as Compared to Neighbors of       C01J Check
PLAT242_ALERT_2_G Low    'MainMol' Ueq as Compared to Neighbors of       C01O Check
PLAT242_ALERT_2_G Low    'MainMol' Ueq as Compared to Neighbors of       C01Q Check
PLAT299_ALERT_4_G Atom Site Occupancy Constrained at ............         0.5 Check
              C2      C3      H2A     H2B     H3A     H3B     H01G    H01H
              H01D    H01F    H01R    H01S    H01P    H01Q
PLAT301_ALERT_3_G Main Residue  Disorder .............(Resd    1)          2% Note
PLAT333_ALERT_2_G Large Aver C6-Ring C-C Dist C00R     -C00Z     .       1.45 Ang.
PLAT335_ALERT_2_G Check Large C6 Ring C-C Range C00R     -C00Z           0.20 Ang.
PLAT398_ALERT_2_G Deviating  C-O-C   Angle From 120 for O00A     .      105.6 Degree
PLAT432_ALERT_2_G Short Inter X...Y Contact  F008     ..C00X     .       2.79 Ang.
                                                  -1+x,y,z  =      1_455 Check
PLAT432_ALERT_2_G Short Inter X...Y Contact  F008     ..C00R     .       2.90 Ang.
                                                  -1+x,y,z  =      1_455 Check
PLAT434_ALERT_2_G Short Inter HL..HL Contact F00O     ..F00P     .       2.83 Ang.
                                                  -1+x,y,z  =      1_455 Check
```

```
PLAT720_ALERT_4_G Number of Unusual/Non-Standard Labels .........         88 Note
              Ag01    Ag02    Ag03    F004    O005    F006    F007    F008
              F009    O00A    N00B    F00C    F00D    F00E    N00F    F00G
              F00H    N00I    F00J    N00K    O00L    N00M    F00N    F00O
              F00P    N00Q    C00R    C00S    C00T    C00U    C00V    F00W
              C00X    C00Y    H00Y    C00Z    C010    H01A    H01B    F011
              C012    C013    F014    C015    C016    C017    C018    C019
              C01A    C01B    H01C    C01C    C01D    H01W    C01E    H01E
              C01F    C01G    H01X    C01H    H01G    H01H    H01D    H01F
              C01I    C01J    F01K    C01L    H01L    C01M    H01I    H01J
              H01K    C01N    H01M    H01N    H01O    C01O    C01P    H01R
              H01S    H01P    H01Q    C01Q    C01R    H01T    H01U    H01V
PLAT791_ALERT_4_G Model has Chirality at C01L       (Sohncke SpGr)          R Verify
PLAT860_ALERT_3_G Number of Least-Squares Restraints ............           7 Note
PLAT883_ALERT_1_G Absent Datum for _atom_sites_solution_primary ..     Please Do !
PLAT910_ALERT_3_G Missing FCF Reflection(s) Below Theta(Min)[Deg]=       3.71 Note
                0  0  2,
PLAT912_ALERT_4_G Missing # of FCF Reflections Above STh/L=  0.600         91 Note
PLAT933_ALERT_2_G Number of HKL-OMIT Records in Embedded .res File         31 Note
              -11  1 12, -10  1  1, -10  2 10, -10  3  2, -10  3  3, -10  3  5,
              -10  3  6, -10  3  7, -10  3 13, -10  4  3, -10  4  4, -10  4  5,
              -10  4  6, -10  4  8, -10  4  9, -10  4 10, -10  4 11, -10  4 12,
              -10  5  6, -10  5  7, -10  5  8, -10  5 15,  -9  6 10,  -9  6 11,
               -9  6 13,  -9  6 17,  -9  7 11,  -9  7 12,  -9  7 13,  -9  7 16,
                9  2  0,
PLAT941_ALERT_3_G Average HKL Measurement Multiplicity ..........        4.0 Low
PLAT969_ALERT_5_G The 'Henn et al.' R-Factor-gap value ..........      1.865 Note
              Predicted wR2: Based on SigI**2  3.72 or SHELX Weight  6.63
PLAT978_ALERT_2_G Number C-C Bonds with Positive Residual Density.          0 Info
```

```
   0 ALERT level A = Most likely a serious problem - resolve or explain
   0 ALERT level B = A potentially serious problem, consider carefully
  11 ALERT level C = Check. Ensure it is not caused by an omission or oversight
  31 ALERT level G = General information/check it is not something unexpected

   2 ALERT type 1 CIF construction/syntax error, inconsistent or missing data
  24 ALERT type 2 Indicator that the structure model may be wrong or deficient
   6 ALERT type 3 Indicator that the structure quality may be low
   9 ALERT type 4 Improvement, methodology, query or suggestion
   1 ALERT type 5 Informative message, check
```

Validation response form

Please find below a validation response form (VRF) that can be filled in and pasted into your CIF.

```
# start Validation Reply Form
_vrf_PLAT042_lcz065
;
PROBLEM: Calc. and Reported MoietyFormula Strings  Differ     Please Check
RESPONSE: ...
;
_vrf_PLAT090_lcz065
;
PROBLEM: Poor Data / Parameter Ratio (Zmax > 18) ........       7.84 Note
RESPONSE: ...
```

```
;
_vrf_PLAT213_lcz065
;
PROBLEM: Atom F00W            has ADP max/min Ratio .....        3.9 prolat
RESPONSE: ...
;
_vrf_PLAT220_lcz065
;
PROBLEM: NonSolvent   Resd 1  C   Ueq(max)/Ueq(min) Range        3.5 Ratio
RESPONSE: ...
;
_vrf_PLAT234_lcz065
;
PROBLEM: Large Hirshfeld Difference C3       --C01H     .       0.16 Ang.
RESPONSE: ...
;
_vrf_PLAT241_lcz065
;
PROBLEM: High   'MainMol' Ueq as Compared to Neighbors of       C01H Check
RESPONSE: ...
;
_vrf_PLAT342_lcz065
;
PROBLEM: Low Bond Precision on  C-C Bonds ..............      0.011 Ang.
RESPONSE: ...
;
_vrf_PLAT369_lcz065
;
PROBLEM: Long   C(sp2)-C(sp2) Bond  C00R     - C00X     .       1.55 Ang.
RESPONSE: ...
;
# end Validation Reply Form
```

It is advisable to attempt to resolve as many as possible of the alerts in all categories. Often the minor alerts point to easily fixed oversights, errors and omissions in your CIF or refinement strategy, so attention to these fine details can be worthwhile. It is up to the individual to critically assess their own results and, if necessary, seek expert advice.

PLATON version of 17/09/2025; check.def file version of 17/09/2025

---

duplicate check

No duplication found

Datablock lcz065 - ellipsoid plot

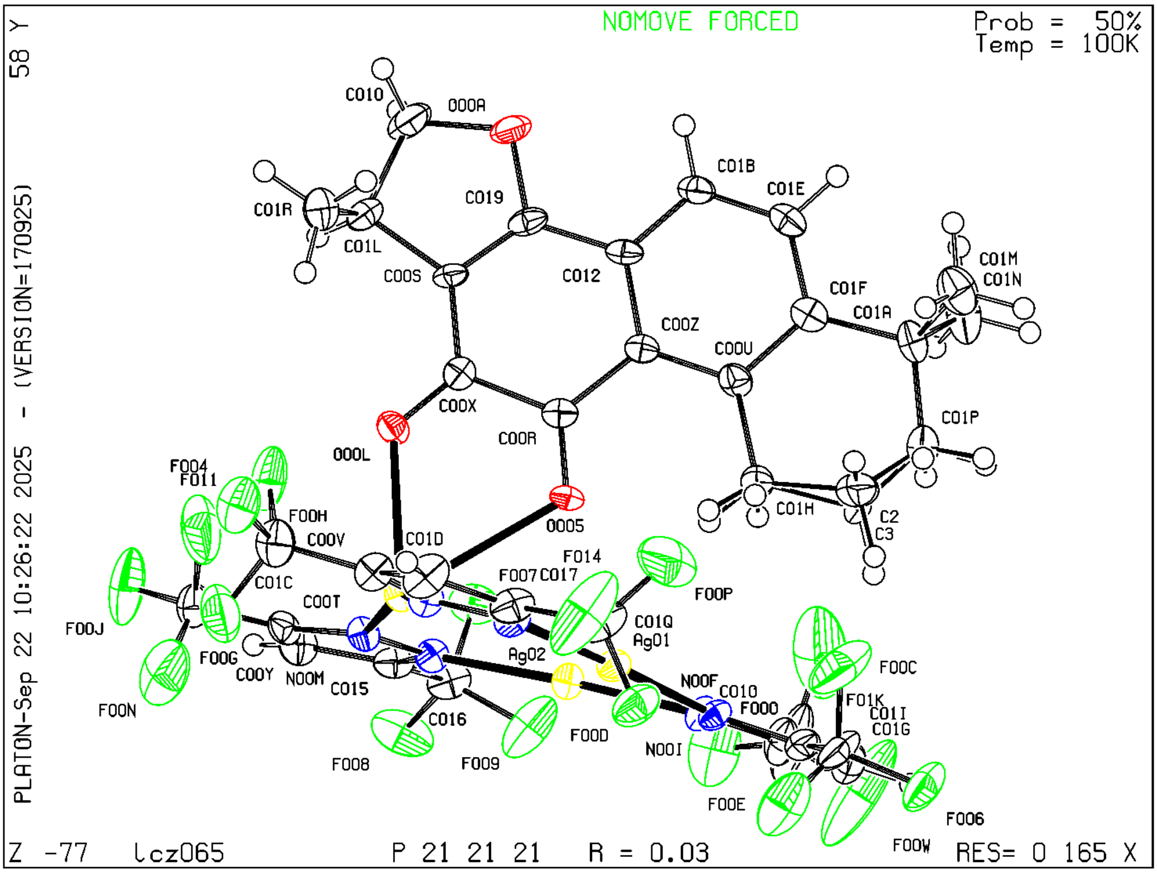

Supplement: Supplementary file 2 — Supporting File 2: anie72462‐sup‐0002‐DataFile.zip. [file ANIE-65-e1218503-s002.zip › supplementary_materials_crystallographic_data submit.zip!/cif_and_checkcif(1-114)/Ag3Pz3_65/Ag3Pz3_65_checkcif.pdf]
